# Supplementary material for: MR Imaging Biomarkers for Clinical Impairment and Disease Progression in Patients with Shoulder Adhesive Capsulitis: A Prospective Study
Source: J Clin Med. 2021 Aug 29;10(17):3882. doi: 10.3390/jcm10173882 (PMC8432015; doi:10.3390/jcm10173882)
Supplement: Supplementary file 1 [file jcm-10-03882-s001.zip › jcm-1348160-supplementary.pdf]

Supplementary Materials:

Table S1: Modified Constant-Murley Score

Parameters	Degree	Points
Pain	None	15
	Mild	10
	Moderate	5
	Severe	0
Activties of daily living	Full work	4
	Full recreation/sport	4
	Unaffected sleep	2
Arm positionning	Up to the waist	4
	Up to the xiphoid	2
	Up to the neck	6
	Up to the top of the head	8
	Above the head	10
Forward elevation	0–30°	0
	31–60°	2
	61–90°	4
	90–120°	6
	121–150°	8
	151–180°	10
Abduction	0–30°	0
	31–60°	2
	61–90°	4
	90–120°	6
	121–150°	8
	151–180°	10
Combined external rotation	Hand behind head, elbow forward	2
	Hand behind head, elbow back	2
	Hand on top of head, elbow forward	2
	Hand on top of head, elbow back	2
	Full elevation from top of head	2
Combined internal rotation	Interscapular region	10
	Inferior tip of scapula	8
	Twelfth rib	6
	Lumbosacral junction	4
	Buttock	2
	Lateral Thigh	0
	Score	x/75
Diurnal Pain	Yes/No	
Night Pain	Yes/No	
Predominance of Night Pain	Yes/No	
Pain duration	No pain	0
	Less than 6 weeks	1
	Between 6 weeks and 3 months	2
	Between 3 and 6 months	3
	Between 6 months and 1 year	4
	Over 1 year	5
